# The Impact of Social Media Marketing on Consumer Engagement in Sustainable Consumption: A Systematic Literature Review

**DOI:** 10.3390/ijerph192416637

**Published:** 2022-12-11

**Authors:** Paweł Bryła, Shuvam Chatterjee, Beata Ciabiada-Bryła

**Affiliations:** 1 Department of International Marketing and Retailing, Faculty of International and Political Studies, University of Lodz, Narutowicza 59a, 90-131 Lodz, Poland; 2 Doctoral School of Social Science, University of Lodz, Matejki 22/26, 90-297 Lodz, Poland; 3 Department of Preventive Medicine, Faculty of Health Sciences, Medical University of Lodz, Zeligowskiego 7/9, 90-752 Lodz, Poland

**Keywords:** consumer engagement, social media marketing, systematic review, bibliometric analysis, relationship marketing, sustainable consumption

## Abstract

Social media have progressed drastically in building successful consumer engagement both in brand building and sustainable consumption. This paper is a review of the articles concerning the influence of social media marketing on consumer engagement in sustainable consumption practices published over the last 8 years. We follow the PRISMA technique as a methodological approach. The review investigates 70 empirical research articles published between 2014 and 2022. A total of 70% of the reviewed articles were published during the last two years. The most influential theories in this field of study are relationship marketing and consumer engagement (16 articles), social exchange (10), and sustainable consumption (8). The most commonly used methods are quantitative (in as many as 61 of the 70 reviewed articles). A careful analysis of the reviewed articles suggests that the tools that are consistently contributing to sustainable consumption are influencer marketing along with creating meaningful content with the right balance of content design, quality, and creativity, as well as the use of emojis. Consumer involvement with a brand relationship quality is key to a sustainable lifestyle. Young individuals with an entrepreneurial vision and a high drive for increased social status demonstrate the highest social media engagement in sustainable consumption.

## 1. Introduction

In today’s world of business, engagement in any form appears to be the buzzword [1]. Consumers expect brands to connect with them more on an emotional level than just selling their products and services. This depicts a shift from a transactional marketing perspective to a more in-demand relationship focus approach [2,3]. Consumer engagement happens to receive major attention from marketers if they think of building a long-term relationship with their consumers, which will help them secure brand awareness [4,5] and loyalty toward their brands [6]. Marketing practitioners across the globe have realized the significant potential of investing time in the digital space considering a variety of social media platforms [7,8]. The same has been certified by the Marketing Science Institute [9], which has included consumer engagement as their top priority for the coming years in delivering top-notch customer value. Bhattacharjee [10] discussed how the digital space comprising social media tools would have projected estimated spending of more than USD 750 billion by 2025. Consumer engagement is a multifaceted approach comprising the cognitive, behavioral, and affective aspects of the brand–consumer relationship [11,12].

Past studies have suggested that it is consumer engagement that acts as the initiator for brands building a long-term relationship with their consumers [13]; often, on most occasions, under its presence, consumers tend to demonstrate a favorable attitude toward the brands as well [14]. Lim et al. [6] have suggested consumer engagement as an emerging topic, which has progressed rapidly over the past decade. Hence, it is important to have an overview of past studies to build up a future trajectory to enrich our understanding of the concepts of consumer engagement in social media marketing. Several reviews have appeared in the literature over the last few years. Some of them were focusing on the implications of the managerial perspective and building a connection through social media from a B2B standpoint [15,16], whereas other reviews focused on the various theories adopted in the literature [17]. Reviews from the domain perspective, such as hospitality and tourism [18], were also apparently visible. Haider et al. [19] discussed the importance of sustainable consumption from the micro, meso, and macro levels to practice a better quality of life by training consumers in thoughtful consumption [20] and providing them with better infrastructural instruments. A systematic review by Fischer et al. [21] guides us on building communication as an integral tool for practicing sustainable consumption.

Epstein [22] discussed sustainable consumption as a consumer’s long-term awareness of consequences to the natural or social environment, often expressed through words such as environmentally friendly or socially friendly consumption behavior [23]. Although this field has developed intensely in recent years, nevertheless, the implementation of sustainable consumption by consumer engagement through social media is still in its nascent state. Green thinkers are individuals with a more conscious approach and responsible intentions and decision making when it comes to environmental issues [24]. De Morais et al. [25] discussed how consumers with selfless concerns for others’ well-being and culture are shaping the motives for sustainable consumption through social media. Consumers engaged in deeper participation in social media are actively trying to promote green buying for sustainable consumption. Kong et al. [26] suggest that high-end brand advertisers on social media should be respectful of consumers’ cultural orientation in building sustainable consumption interaction. Xia et al. [27] suggested how sustainable resource management by encouraging environmental innovation could contribute to improved performance for sustainable corporations through social media networks. Finally, Zafar et al. [28] suggest how a personalized advertisements approach attracts consumers to a sustainable purchase decision in social media networks.

To understand the popularity of social media as an effective tool to build consumer engagement in the sustainable consumption environment, catching the diversity and depth of the current research in this genre, a more detailed systematic review combining the future untapped research directions along with the research questions to clarify those dimensions is of utmost need. This review tries to bridge this gap by discussing the themes that emerged along with the characteristics portrayed over the last eight years, thus paving the way for future research questions and research directions for social media marketing researchers involved in consumer engagement in the social media brand community.

This work is an illustrative overview of articles on the social media brand communities involving consumer engagement with a special focus on (1) the various research methodological approaches and variables identified over this span of eight years, (2) research theories supporting previous research, and (3) future research directions along with research questions to assist social media marketing scholars in conducting fruitful and relevant research in this field. 

This review provides insights into several research questions:

1. What are the characteristics of the recent literature on social media consumer engagement in sustainable consumption in terms of theories, contexts, and methods?

2. How was consumer engagement in social media brand communities operationalized in research models (independent or dependent variable, control, or moderator)?

3. What further investigations could be conducted by scholars into consumer engagement and building sustainable consumption through social media?

The remainder of the paper is organized as follows. The following section presents the literature review methodology. Subsequent sections include a substantive analysis of the research studies included in this review and discussion, including limitations, future research directions, and a conclusion.

## 2. Materials and Methods

The approach followed for this study is a meta-textual review further allowing the identification and extraction of the pertinent information on subjects of relevance from published research and assessing the literature [29]. This approach has the following goals:

(i) To assess relevant and quality articles focused on consumer engagement with a direct intervention with social media marketing.

(ii) To formulate an integrative framework providing a holistic understanding of the impact of social media marketing on consumer engagement in sustainable consumption.

(iii) To identify the research gaps in the literature and provide future research directions.

A systematic literature review is evidence of the previous literature that accurately and reliably analyzes the quality of peer-reviewed journals following some preferred reporting items and consisting of a meta-analytical structure (PRISMA) [30]. PRISMA is a structured review protocol, which provides a four-phase flow diagram representing the sample identification for screening and then for eligibility testing and the final demonstration of the studies included in the review. The logic for choosing PRISMA lies in its comprehensiveness and its potential to provide more consistency across its reviews. To conduct this review, four steps were followed, namely, (1) establishing the inclusion–exclusion criteria for study selection, (2) identifying relevant quality studies, (3) evaluating the literature, and (4) reporting the findings.

The sample search strategy and identification involve three activities, namely, (a) searching appropriate keywords, (b) assessing the relevance, and (c) assessing the quality.

### 2.1. Assessing Appropriateness of the Search Keywords

The data search was executed using a prominent multidisciplinary database of peer-reviewed research literature, Web of Science. Li et al. [31] discussed the usability of this database gaining increasing popularity in scientific instruments across countries and knowledge domains. 

**Figure 1:**
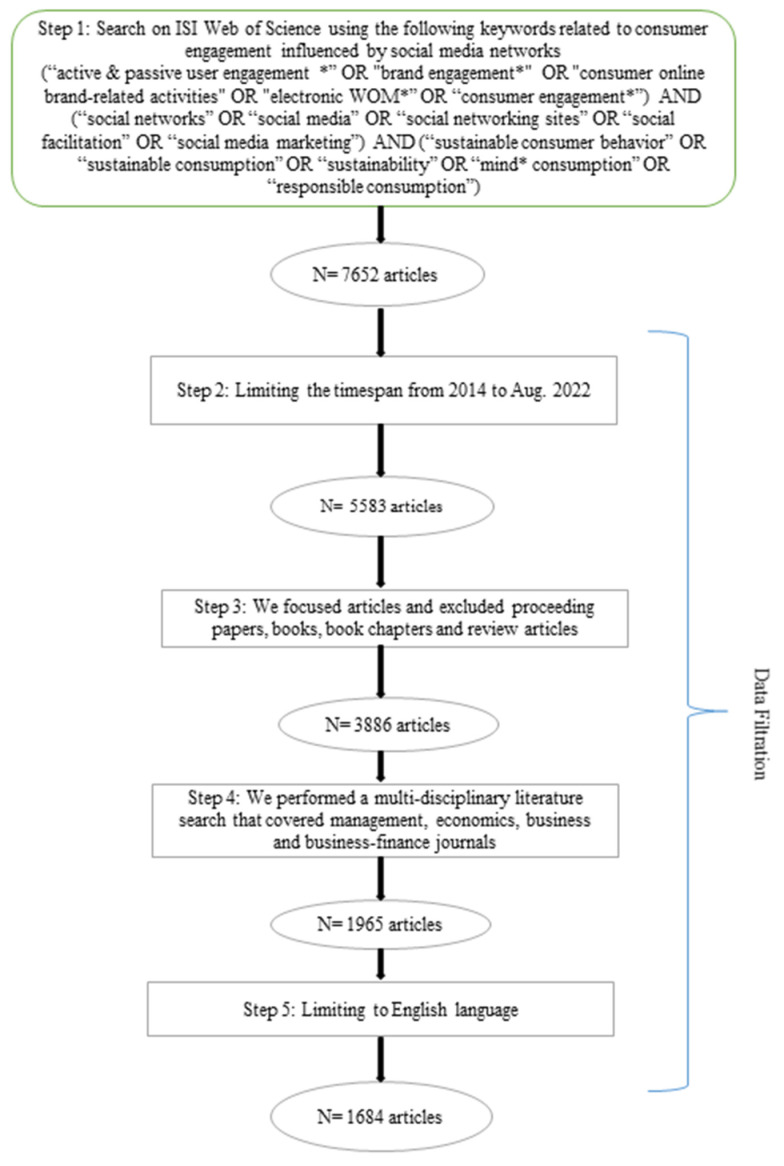
Identification of initial inclusion criteria for articles in this review. Note: * in the search string denotes that different word endings following this symbol are included in the search.

The search strings were created by regrouping the chosen keywords into three specific categories. The first category covers terms representing consumer engagement, and the second category is formulated using the influence of social media marketing. Finally, the third category investigates the sustainable consumption category. The search strings are presented in Figure 1.

The keywords were mainly searched in the titles, abstracts, and/or keyword sections, and consequently, 7652 articles were identified from the search process. Considering the appropriateness of the journals and limiting the time according to the inclusion and exclusion criteria set, a total of 1684 articles were identified, as shown in Figure 1.

### 2.2. Assessing Relevance

Initial sorting of the articles’ titles and abstracts led to the exclusion of articles that did not focus on consumer engagement and social media marketing in the context of sustainable consumption. We excluded papers on the grounds of trade publications, editorial handbooks, overlapping studies in close contexts of consumer engagement, and dissertations, to ensure further homogeneity.

The rationale for the sample considering research papers after 2014 is manifold. First, the study tries to acknowledge the recent trends of methodologies and to understand the recent shift in research methods and techniques. Moreover, the context of studies has significantly varied from understanding consumer engagement in brands to hospitality, to influencers and their social media activities involving consumer engagement. Hence, this present study would primarily focus on understanding these pattern shifts.

Finally, 265 articles were selected for deeper reading, allowing us to discard 88 working papers. After these steps, the resulting sample consisted of 177 articles.

### 2.3. Assessing Quality

**Figure 2:**
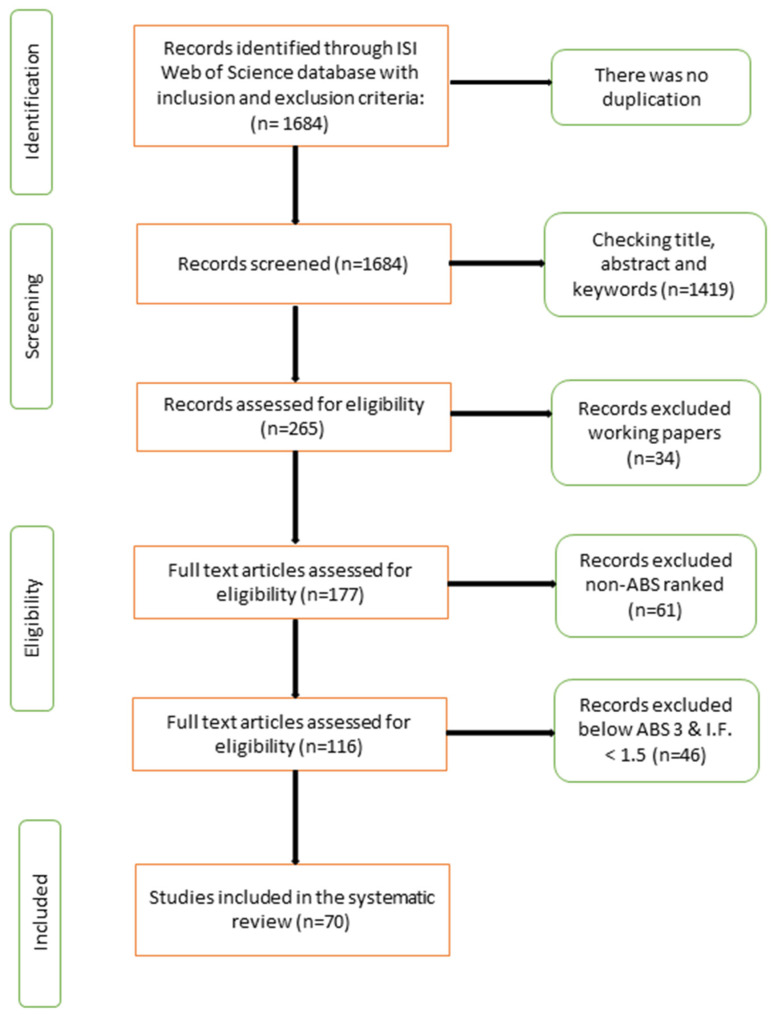
Flowchart of the study selection process regarding relevance and quality of the initially selected studies.

Many a time, an article seems relevant, but it might lack quality. Hence, a consistent focus on peer-reviewed and high-quality journals was chosen along with journal ranking criteria based on the Association of Business Studies (ABS) Journal Quality Guide and only included top journals ranked as 4*, 4, and 3 to generate high-quality articles. This refinement process led to inclusion of 70 articles in this systematic review (Figure 2). A study by Mingers and Yang [32] suggested from a sample of over four hundred research articles from the ABS journal ranking list that the standard mean impact factor was around 1.25. Hence, for this study, we accommodated articles with an IF of at least 1.5 and above. Rowlinson et al. [33] suggested that ABS-ranked articles (above 3) defined quality levels as internationally excellent in terms of originality and rigor, which set up the base for our study to have articles listed in ABS 3 and above.

## 3. General Overview of Articles Included in This Review

### 3.1. Publication Trends

**Figure 3:**
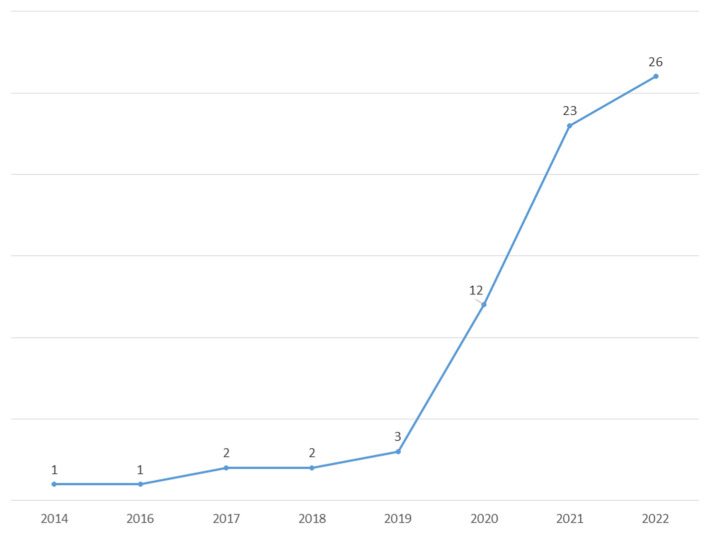
The number of articles included in this review by the year of publication.

The year-wise distribution of articles presented in Figure 3 witnesses a sharp rise in the number of articles on consumer engagement within the context of social media in the last two years (i.e., 2021 and 2022). This implies that consumer engagement is gaining popularity and witnessing a growth phase in terms of the number of articles published in the area. In the last two years, the number of published articles increased so much that more than 65% of the total studies were published in the last two years.

### 3.2. Classification of Articles

To measure the progress of the impact of social media marketing on consumer engagement, we classified the empirical studies [16] into either qualitative (which bring out results where the primary data points are non-numeric) or quantitative or mixed methods. Out of the 70 articles studied, we figured out that only 7 studies adopted a qualitative path, while 2 studies adopted a mixed method approach. However, most of the articles were based on research through a quantitative approach (61 articles).

## 4. Meta-Textual Method

### 4.1. Theories

Consumer engagement in the context of social media has gained momentum across various theoretical contexts from various disciplines to showcase its effects. The study identifies 57 studies that have employed at least one theory. Here, we discuss the most applied theories in consumer engagement (Table 1).

#### 4.1.1. Relationship Marketing and Consumer Engagement

This section deals with the combined interrelated theories of relationship marketing and consumer engagement, which account for 25% of the total studies (16 studies). According to Pansari and Kumar [76], consumer engagement has shaped out to be one of the crucial elements in contemporary marketing, with its direct effects on relationship marketing. Further, they discuss that emotion and satisfaction are the main pillars of consumer engagement. Moreover, they conclude that engagements can only be nurtured if consumers tend to show belongingness toward the brand and form relationships in due process. Gómez et al. [34] suggest that consumer engagement is stronger with social media brand engagement than just brand communication. Ma et al. [12] suggest how strong brand engagement in the form of posts, tweets, and continuous interaction with consumers contributes to relationship building impacting the consumer’s behavioral, cognitive, and emotional engagement.

#### 4.1.2. Social Exchange

The social exchange theory, which enlists psychology, sociology, and economics [77], has also been witnessed among consumer engagement theories (16%, 10 studies). The essence of this theory is to understand the focus for consumers to get involved through social media marketing [78]. According to Zhao and Chen [42], consumers tend to develop a more psychological bond when they are satisfied with the brand and its involvement in marketing. Consumers derive perceived benefits and satisfaction responses by engaging in social media activities [45]. A study by Kim and Baek [47] suggests the impact of influencers in engaging consumers and building a network of relationships.

#### 4.1.3. Sustainable Consumption

The sustainability theory is highly visible in online consumer engagement through social media in the recent past, as consumers tend to become extremely aware of their purchases having an environmental impact. This pushes the demand for increased green sustainable brands [79]. Kong et al. [26] discussed how effective sustainable communication is in selling luxury products, keeping the cultural orientation of the consumers in mind at the same time. Nekmahmud et al. [53] suggested that online consumers’ need to engage with a positive attitude towards green products, which would have a strong association with sustainable consumption. Further, socio-environmental and socio-economic thoughts play a crucial role in building sustainable brand performance [27]. Zafar et al. [28] attempt to understand the importance of crafted personalized advertisements playing a significant role in consumers’ sustainable purchase intentions.

#### 4.1.4. Uses and Gratification

Katz et al. [80] discussed the uses and gratification theory to understand how communication occurs through mass media. This theory concentrates on understanding users’ selection of media based on their goals to cater to specific needs. With the invention of social media, this theory now focuses on understanding the user’s choice and use of the internet. The theory has been consistently used in consumer engagement behavior across social media (seven studies, 10%). The studies talk about consumers’ engagement in participation in social media, including the cognitive and social benefits along with personal achievements in having pleasurable experiences derived from social media interactions. Bailey et al. [59] discuss consumer socialization motivation and participation in social media engagement, which would yield results in achieving brand and marketing goals [60].

#### 4.1.5. Other Theories

In addition to the theories discussed, the study also explains specific behavior, which is categorized in “other theories”, which account for 25% of the studies (16 studies). For example, Liu et al. [75] discuss the trust transfer theory, where consumer engagement plays a significant role in brand trust. Additionally, Lourenço et al. [74] introduced the expectancy theory, which underlines the consumer engagement dimension operational scales for measuring the level of consumer engagement.

### 4.2. Context

This section discusses the countries involved in the analyzed sample. The findings indicate that Europe is the biggest contributor to this study, with 31 of the 70 studies (44%). This reflects that the European countries, mainly the UK, France, Austria, Belgium, and the Netherlands, dominate the papers related to consumer engagement in sustainable consumption within the social media marketing context. Asia, surprisingly, is the second biggest contributor to this research stream (25 studies, 35% of the total empirical papers studied), followed by the USA—19 studies (27%)—and other countries. It is noted that, unlike social science, the contexts are predominantly set in the more emerging Asian market. The feasible logic is the extensive economic and technological advancements witnessed in the Asian market in the last eight years. The study also finds that there will be ample scope for future researchers in the context of consumer engagement through social media in the South American market. Emerging markets, such as Brazil, did not showcase enough contribution in this domain of study. Hence, future research should focus on these markets. Additionally, the researchers would advise future researchers in this field to focus on cross-country consumer engagements in social media; culture would play a significant role in such studies (Table 2).

### 4.3. Methods

In this section, we will discuss the articles reviewed through the prism of the research approaches and analytical techniques adopted to assess the relationships investigated in consumer engagement research. Table 3 and Table 4 demonstrate the data collection techniques and analysis techniques used in consumer engagement, respectively. Surveys are the most used quantitative method. Other methods encountered are content analysis and latent profile analysis. Concerning data analysis techniques, structural equation modeling (SEM) is the most used research tool, accounting for 42% of the total quantitative studies, followed by confirmatory factor analysis (CFA), accounting for 37% of the studies. However, it is to be noted that 10% of the studies in consumer engagement have adopted a combination of qualitative methods, such as in-depth interviews, observational research, netnography, and Google Vision AI. We observed the emergence of netnography as a methodological tool, which is a refined version of ethnographic research occurring in social media communities [68]. Most of the studies initiated conducted surveys through online and social media platforms.

We believe that consumer engagement studies would benefit immensely from conducting more longitudinal studies testing relationships over a period of time. Additionally, there is a lack of studies picking up the experimental method approach to understand a more trusted consumer engagement on social media platforms. Our research validates a greater scope of a mixed-method approach for conducting studies in consumer engagement.

## 5. Variables Used in the Reviewed Research Studies

This section reviews the various independent, moderating, control, and dependent variables in consumer engagement studies influenced by social media marketing and their associated relationships that were tested to unfold certain phenomena concerning these variables (see Table 5).

### 5.1. Independent Variables

The independent variables include cognitive and affective states (8 articles, 12% of the study), their relationship with brands, and consumer engagement (14 articles, 22% of studies) in the tune of social media marketing. The various mental states as demonstrated by consumers include perceived benefits from the brand and behavioral outcomes in building value co-creation and research integration. Consumer-related variables contribute to the understanding of engagement through interaction, advocacy, and connecting with the brand and trust. Social-media-related variables try to test the strength of attachment, having faith in social media channels, and the various follow-up techniques effectively used (tweet reposts, likes, comments) to build consumer engagement. Finally, brand-related variables try to focus on building consumer appeal and brand engagement activities, developing persuasiveness and brand trust, and enriching the brand’s global identity.

### 5.2. Dependent Variables

Our investigation of the dependent variables reveals that most of the studies focus on intentional or behavioral consumer engagement and relationship-based outcomes as well. The intentional and behavioral outcome validates consumers’ word of mouth, feedback, and recommendations along with participation in community engagements. The focus also lies in analyzing social media and brand marketers’ posts from an emotional perspective. It also judges the purchase intention of consumers. The relationship-based outcomes deal with the various engagement activities consumers and brands perform, such as frequent likes and comments of the posts along with sharing them on social media networks. There are then consumer-related variables focusing on the attitude and purchase intentions of consumers, with an overall brand experience, which finally leads to purchase decisions for consumers as well. Finally, consumer-related variables leading to sustainable consumption contribute to green buying intentions, a clear psychological state of well-being, being thought of as an environmental activist, and making a sustainable purchase decision.

### 5.3. Control Variables

Our research on control variables comprises mainly brand or marketer-related and consumer-related control variables. The consumer-related variables discuss the demographic origin of the consumer along with analyzing his/her activities on the networking sites. Additionally, visual perceptions, the timing of posts, and brand familiarity with social networking sites play a crucial role. The brand/marketer-related variables focus on building a buzz about their products and services, thereby maintaining the brand community engagement along with building favorable brand attachments.

### 5.4. Moderating Variables

Finally, the moderating variables consist of consumer-related and brand-related variables. The consumer-related variables come from culture playing a significant role in consumers’ engagement with the brand over social media, while the brand-related variables include the topic and modality of the posts in social media networks.

## 6. Discussion

### 6.1. Limitations

One of the limitations is related to the extremely fast changing social media landscape. Every high-quality indexed journal approves a research paper after considerable time spent by the reviewers understanding the paper’s quality, rigor, and contribution to the research community. Hence, during that review time, further developments can occur in the field of consumer engagement under social media influence, thereby creating a gap where the present researchers fail to accommodate the most recent articles. Second, this review followed strict guidelines to ensure a stringent process of selecting journal articles [86,87]. Hence, because of narrowing down the search criteria to accommodate articles complying with consumer engagement in a social media context, the review might have missed overlapping or close concepts in the literature, such as consumer engagement in a B2B context [16] or interactions in social CRM [88]. Third, social media, if not used effectively by organization salespersons, can often become a tool for exploitation, thereby resulting in consumers interpreting information and communication messages inappropriately. Fourth, “technostress”, as studied by Tarafdar et al. [89], could lead to stress due to spending excessive screen time on social networking sites (Facebook, Twitter, and Instagram) and further contribute to improper time management skills by both firm employees and consumers.

### 6.2. Future Research Directions

A direction for future research is an important lookout for systematic reviews [86]. Based on our review of the findings of research conducted on consumer engagement in social media networks, we noted multiple channels where we would highly encourage future research to occur. Researchers have made noteworthy progress in understanding the role of social media networks in communicating information in the business market. However, Maier et al. [90] discussed that salespersons loaded with excessive information may experience a feeling of discomfort. Hence, future research should follow the direction of understanding the optimum information chain, which would not create fatigue for the message recipients.

Second, it would be interesting to find out to what extent cross-country cultural differences influence the functioning of consumer engagement in social media networks with the use of the Hofstede [91] model and redefining the marketing strategies ensuring societal well-being by executing mindful consumption [19].

Third, it will be interesting to understand the consumer sentiment toward social media networks in building engagement in green consumption. Researchers could deploy analytical methods to forecast future consumer engagement in social media networks, measuring constructs such as the strength of the attachment and the total revenues generated for the firm through liking and sharing of tweets. Finally, future research could be carried out to understand how consumer engagement in sustainable consumption could be stimulated in social media networks in developing countries, such as Brazil, Indonesia, and India. This is because countries such as India and Brazil are dominating social networking sites [92], and hence, diversified fresh research looking for a varied research focus is much needed to understand the growth of social-media-based consumer engagement strategies.

## 7. Conclusions

This study constitutes an attempt to assess the state of the art in the hyper-dynamic field of social media consumer engagement in sustainable consumption. We analyzed research articles that examined the role of social media networks in engaging consumers to become attracted to a sustainable brand or product. We believe that this review will enable the scholarly community to initiate and conduct relevant research in this vital emerging research area.

According to our results, the investigated research area is gaining a rapidly increasing interest in the scientific community, as evidenced by the number of studies published. A total of 49 articles included in this review were published during the last two years. Twelve appeared in 2020 and only nine in the period of 2014–2019.

Most of the reviewed studies have been published in Europe (44%), followed by Asia (35%), and the USA (27%).

The most influential theories in this field of study are relationship marketing and consumer engagement (16 articles), social exchange (10), and sustainable consumption (8).

The most commonly used methods are quantitative (in as many as 61 of the 70 reviewed articles). The prevalent data analysis techniques are SEM (28 studies), CFA/EFA (23), and various regression models (16).

A careful analysis of the reviewed articles suggests that the tools that are consistently contributing to sustainable consumption are influencer marketing [73] along with creating meaningful content with the right balance of content design, quality, and creativity. Moreover, the meaningful use of emojis [69] is gaining immense popularity among social media practitioners for building sustainable marketing consumption through a rapid increase in likes and comments in the posts [44] and an array of text characteristics with emojis [64].

The review led to the conclusion that consistent and disciplined consumer involvement [49] with a steady brand relationship quality [34] is key to a sustainable lifestyle and behavior contributing to sustainable consumption.

This systematic review is a work that draws attention to the consumer segments, which are prone to adopting new technologies [48]. Young individuals [51] with an entrepreneurial vision [54] and a high drive for increased social status [25] are seen as actively involved in social media engagement in sustainable consumption. One very important observation that came out from this review is that consumers’ attitudes and purchase intentions toward social-media-based brand marketing activities depend largely on the consumers’ generation, and hence, all activities need to be fine-tuned respecting and understanding the age profile of the target audience [82].

## Figures and Tables

**Table 1:** Theories used in studies on consumer engagement in social media in the context of sustainable consumption.

Theories	No. of Articles	Examples
Relationship marketing and consumer engagement	16	[7,12,34,35,36,37,38,39,40,41]
Social exchange	10	[42,43,44,45,46,47,48,49,50]
Sustainable consumption	8	[25,26,27,28,51,52,53,54]
Uses and gratification	7	[55,56,57,58,59,60,61]
Other theories	16	[35,62,63,64,65,66,67,68,69,70,71,72,73,74,75]

**Table 2:** Articles included in the review by country.

Country	No. of Articles
USA	18
China	11
United Kingdom	11
Austria	4
Belgium	4
Canada	4
France	3
Germany	3
Netherlands	3
Spain	3
Australia	2
Hungary	2
India	2
New Zealand	2
Poland	2
Portugal	2
Taiwan	2
Thailand	2
Chile	1
Denmark	1
Egypt	1
Ghana	1
Greece	1
Jordan	1
Korea	1
Malaysia	1
Norway	1
Saudi Arabia	1
South Africa	1
UAE	1
Vietnam	1

**Table 3:** Methodologies adopted in consumer engagement research.

Type of Article	No. of Studies	Examples
Quantitative	61	[7,12,26,28,34,35,38,39,40,42,44,47,49,52,54,55,56,57,63,64,65,74,81,82,83]
Qualitative	7	[36,37,45,68,84]
Mixed	2	[66,85]

**Table 4:** Data analysis techniques adopted in the reviewed articles.

Data Analysis Techniques	No. of Articles
PLS Structural Equation Modeling/SEM	28
CFA/EFA	23
Regression, OLS Regression, Multi-level mixed effects regression, Ctree Regression, Panel Vector Auto regression Method	16
Correlation	8
Content analysis	4
In-depth interview	3
Observational research	3
ANCOVA/ANOVA	3
Chi-square automatic interaction detection analysis (CHAID)	2
Netnography	2
Smart PLS	1
Sentiment analysis	1
Kruskal–Wallis test	1
Principal Components Analysis	1
Data Envelopment Analysis (DEA)	1
Google Vision AI	1
Cluster Analysis	1
Necessary Condition Analysis	1
Mediation Analysis	1
Latent Profile Analysis	1

**Table 5:** Variables investigated in social media consumer engagement research in the context of sustainable consumption.

Variables	No. of Studies	Examples	Contributing Theory
Independent variables			
Consumer-related variables	22	Interaction, advocacy and connection, message throw, consumption and creation, use of first person singular pronouns in consumer engagement, perception of the user, consumer trust, perceived benefits; sensory and behavioral outcome, value co-creation and research integration, hedonic value and ethical value motivations	Customer engagement theory, uses and gratification theory
Brand/marketer-related variables	20	consumer appeal, marketer-generated dialogs, brand engagement behavior, post information and post interactivity, brand gratitude, loyalty, perceived quality, message persuasiveness, brand trust, advertising, brand’s global identity, brand post characteristics	NA
Social-media (SM)-related variables	18	strength of attachment to SM channels, communication, attitude, awareness, loyalty, user’s perceived value and satisfaction, SM influence, SM interactions, likes, follows and tweets, post length, language complexity, text characteristics, tweet readability, tweet frequency	Socialization theory/network theory
Dependent variables			
Consumer engagement (intentional/behavioral)	21	WOM/eWOM, feedback, recommendations, conversations, endorsements, participation, community engagement, revenue, cognitive and emotional perspective, uncovering and cultivating posts, affection and cognitive processing, purchase intentions	Customer engagement theory
Relationship-based outcomes	11	likes, comments, and shares of the posts, a sense of being attracted to others, feeling at ease	Relationship marketing, social identity
Brand/marketer-related variables	11	stakeholder engagement, brand intimacy, value cocreation, brand performance, like and retweet, brand trust	NA
Consumer-related variables leading to sustainable consumption	8	Green buying, psychological state of well-being, focused on an issue, environmental activism	Sustainable consumption
Other consumer-related variables	5	attitude, purchase intentions, brand experience, purchase decision, user’s global identity	NA
Social media engagement	4	likes, comments, story replies, profile checks, shares on Instagram, influence on m-banking acceptance	NA
Control variables			
Consumer-related variables	7	country of origin, posting experience, age, gender, visual perceptions on Instagram, timing of posts, customer trust, brand familiarity, network size	Commitment trust theory
Brand followers, exclusivity	3	brand community engagement, brand attachment	NA
Brand outcome with time	2	release time	NA
Moderating variables			
Brand/marketer-related variables	2	topic and modality of posts	NA
Consumer-related variables	2	cultural differences, consumer personal dimensions, fun dimensions	NA
Social media context	1	media richness, content trustworthiness	NA

## Data Availability

Not applicable.
